# Herpes Proctitis in Men Mimicking Rectal Adenocarcinoma: Two Cases of an Easily Overlooked Diagnosis in the Proximal Rectum

**DOI:** 10.1155/2023/6947960

**Published:** 2023-07-27

**Authors:** Jing Sun, Reenu Malhotra, Lakshmi Ananthakrishnan, Purva Gopal

**Affiliations:** ^1^Department of Pathology, University of Texas Southwestern Medical Center, Dallas, TX, USA; ^2^Propath, Dallas, TX, USA; ^3^Department of Radiology, University of Texas Southwestern Medical Center, Dallas, TX, USA

## Abstract

We describe two cases of rectal herpes simplex virus (HSV) infection in men that clinically mimicked rectal adenocarcinoma. Herpes infection in this location more commonly presents as an anal mass with viral inclusions in squamous epithelial cells. We report these cases to increase awareness of the unusual presentation as a proximal rectal mass with viral inclusions in endothelial cell nuclei. One patient was HIV-positive, and the other one had a history of having sex with men (MSM). Both patients had a thickened rectal wall with prominent lymphadenopathy on computed tomography (CT) scan, suspecting for malignancy. Biopsy showed abundant granulation tissue, necrosis, and inflammatory infiltrate composed predominantly of lymphocytes with admixed numerous plasma cells, eosinophils, and neutrophils. Rare granulation tissue vessels were lined by endothelial cells with nuclear molding and chromatin margination, and nuclei that were positive for HSV immunohistochemistry (IHC). One patient had confirmatory viral culture from biopsy of the ulcerated rectal mass. Both patients had symptom resolution following treatment for HSV. HSV should be considered in the differential diagnosis of rectal inflammatory masses, particularly in immunocompromised, HIV-positive, and MSM patients.

## 1. Introduction

Herpes simplex virus (HSV) is a sexually transmitted infection (STI) which has a high prevalence among human immunodeficiency virus- (HIV-) positive patients and in men who have sex with men (MSM) [1]. The Centers of Disease Control report the seroprevalence of HSV-1 and HSV-2 among 14-49 year-olds is 47.8% and 11.9%, respectively [1]. The seroprevalence of HSV is particularly high in the MSM population [2]. In particular, the seroprevalence of HSV-2 infection in MSM is 18.4% as compared to 11.5% among other men [2]. Although not exclusively, HSV-2 is more commonly known to manifest in the anorectal region as painful, itchy, vesicular, and sometimes ulcerated skin lesions which are occasionally described as nodular, mimicking either condyloma, or other anal/perianal tumor [3–5]. Additionally, HSV infection has been reported to present as a pseudotumoral lesion in other sites including the genital tract, conjunctiva, nasal cavity, and bronchus [5–8].

It is important to consider sexually transmitted infections (STI) as a part of the differential diagnosis of rectal inflammatory mass lesions, particularly in immunocompromised, MSM, and HIV-positive patients. HSV infection is commonly seen and considered in the differential diagnosis of anal/perianal skin lesions rather than in the proximal rectum. There are few detailed reports/descriptions of mass-forming HSV proctitis involving the proximal rectum in the surgical pathology literature. Therefore, we present the clinical and histologic features of HSV proctitis in two male patients, clinically mimicking rectal adenocarcinoma, who presented with ulcerated rectal masses several centimeters proximal to the anus, with prominent associated inguinal and iliac lymphadenopathy on imaging procedures.

## 2. Case 1

**Figure 1:**
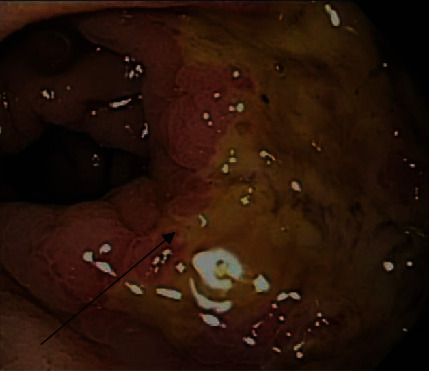
Endoscopic image of thickened, ulcerated mucosa involving 75% of the rectal circumference in case 1 (arrow).

A 22-year-old MSM with no significant past medical history presented to the emergency department for severe rectal pain, painful and bloody diarrhea, difficulty with urination, fever, and a 10-pound weight loss in the prior month. A physical examination revealed suprapubic tenderness. A digital rectal examination was deferred due to pain. Laboratory testing for *Neisseria gonorrhea, Chlamydia trachomatis*, syphilis, and HIV was negative. A CT scan showed proctitis and adjacent diffuse lymphadenopathy, highly suggestive of rectal carcinoma. There was a continuous area of nonbleeding ulcerated, thickened mucosa involving three quarters of the rectal circumference, 12 cm proximal to the anal verge (Figure 1) on flexible sigmoidoscopy. Biopsies were obtained from the ulcers and ulcer edge for histology and viral culture.

**Figure 2:**
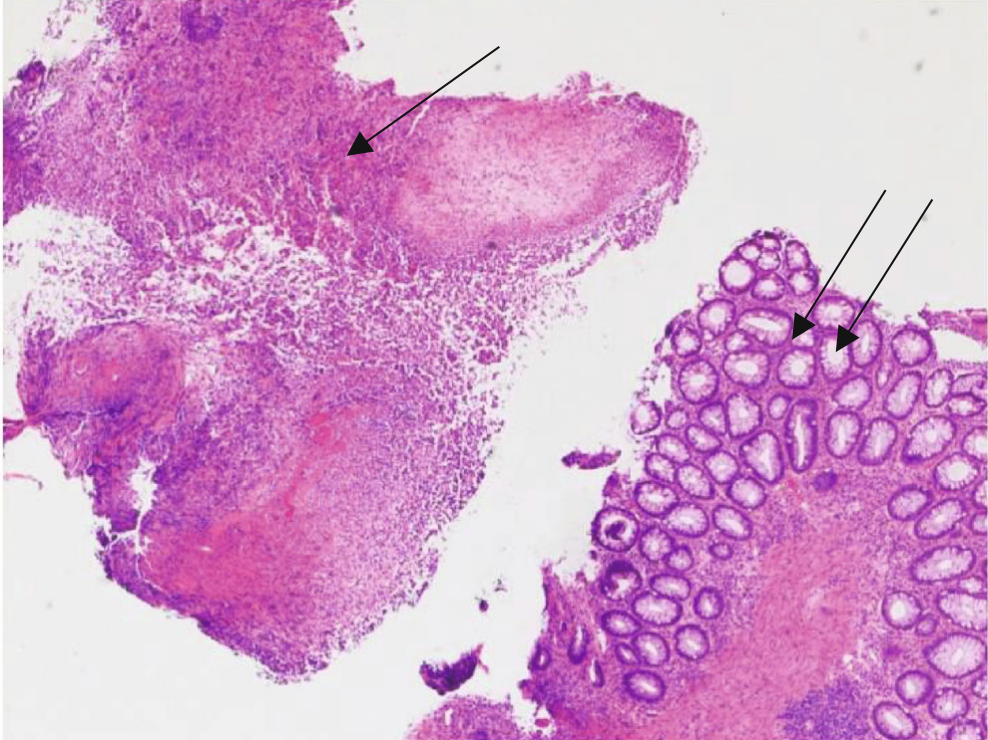
Fragment of ulcerated and necrotic rectal mucosa (arrow) adjacent to a fragment of rectal mucosa with prolapse and reactive epithelial changes in case 1 (double arrow; H&E 10x).

**Figure 3:**
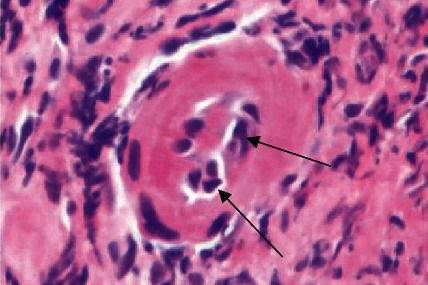
Endothelial cell nuclei of a vessel within granulation tissue with multinucleation, margination of chromatin, and molding in case 1 (arrows; H&E 60x).

**Figure 4:**
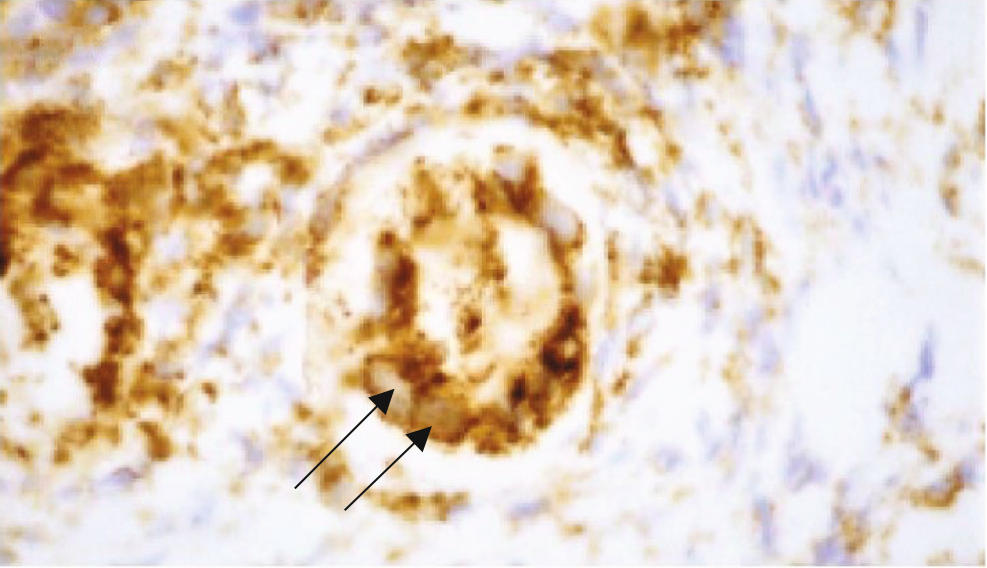
CD31 immunohistochemical stain highlighting endothelial cells of a granulation tissue vessel with enlarged endothelial nuclei containing viral inclusions in case 1 (arrows; 60x).

**Figure 5:**
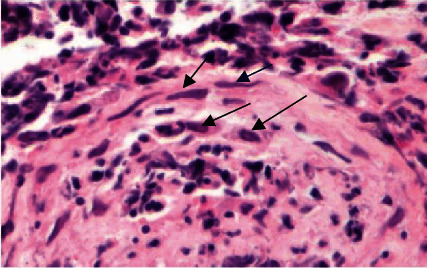
Cluster of fibroblasts within granulation tissue with viral cytopathic effect including nuclear margination of chromatin in case 1 (arrows; H&E 60x).

**Figure 6:**
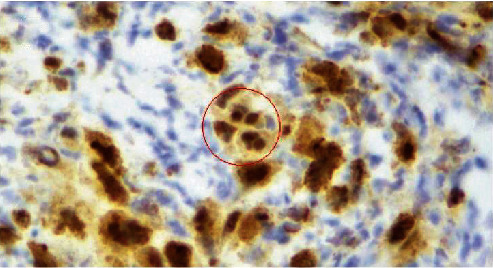
Herpes Simplex Virus immunohistochemical stain highlighting viral inclusions within granulation tissue fibroblast nuclei and within endothelial cell nuclei lining a vessel (red circle) in case 1 (40x).

On hematoxylin and eosin (H&E) slides, the rectal biopsy was primarily composed of granulation tissue suggestive of ulceration, with extensive necrosis and acute inflammatory exudate. The rectal mucosa present had reactive epithelial changes in a background of mucosal prolapse (Figure 2). Within the granulation tissue, there was a prominent mixed inflammatory infiltrate composed predominantly of lymphocytes, with abundant admixed plasma cells, eosinophils, and neutrophils. The endothelial cell nuclei lining rare vessels within the granulation tissue had a ground glass appearance with chromatin margination and molding (Figures 3 and 4), and similar viral inclusions were also seen in fibroblast nuclei adjacent to the vessels (Figure 5). HSV immunohistochemical stain (IHC) highlighted viral inclusions within endothelial cell and fibroblast nuclei (Figure 6). Crypt epithelial cell nuclei were negative for HSV IHC. CMV immunohistochemical stain was negative. There was no perianal squamous epithelium in the biopsy. Rectal viral culture was positive for HSV-2, and HSV PCR was positive from the rectum specimen and blood. The patient completed antiviral treatment with acyclovir and reported symptom resolution.

## 3. Case 2

**Figure 7:**
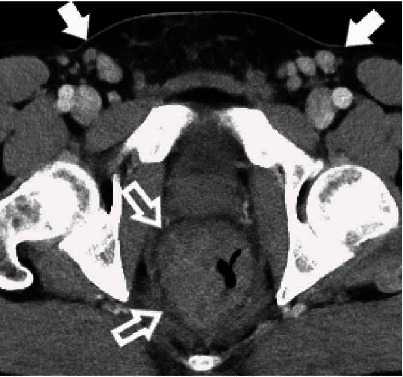
Contrast-enhanced CT in case 2 shows marked eccentric thickening of the rectal wall (open arrows) with adjacent superior rectal (not shown), external iliac (not shown), and bilateral inguinal lymphadenopathy (solid arrows). The degree of eccentric wall thickening made malignancy high on the differential diagnosis.

A 45-year-old HIV-positive man undergoing HIV treatment presented with two months of progressive rectal pain. Associated symptoms included a 10-pound weight loss, constipation, occasional hematochezia, and intermittent urgency with urination. HIV viral load was undetectable, and a CD4 count was not performed at this time. The patient denied MSM. Recent testing for *Neisseria gonorrhea, Chlamydia trachomatis*, and syphilis was negative. The patient had tenderness on digital rectal examination. A pelvic CT-scan showed a long segment of marked rectal wall thickening with an adjacent 4.7 cm thick-walled perirectal air-fluid collection and prominent pelvic lymphadenopathy (Figure 7). The radiologic differential diagnosis included a rectal neoplasm. Endoscopy findings included a large posterior ulcer above the dentate line and a thickened rectal wall, of which multiple biopsies were taken.

**Figure 8:**
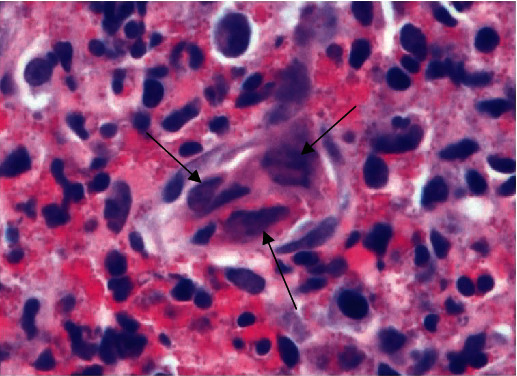
Endothelial cell nuclei of a vessel within granulation tissue with multinucleation, margination of chromatin, and molding in a background of a mixed inflammatory infiltrate composed of lymphocytes, plasma cells, neutrophils, and abundant eosinophils in case 2 (arrows; H&E 60x).

The H&E-stained sections of rectal biopsy in this case were predominantly composed of ulcer and granulation tissue with necrosis and prominent mixed inflammatory infiltrate similar to case 1. Endothelial cell nuclei within rare granulation tissue vessels had viral-cytopathic effect including chromatin margination and molding (Figure 8). These endothelial cell nuclei were positive for HSV IHC and negative for CMV IHC. The patient was temporarily lost to follow-up following the diagnosis. He returned after 5 months with persistent symptoms. A repeat pelvic CT scan and magnetic resonance imaging (MRI) again demonstrated rectal wall thickening prominent associated inguinal and iliac lymphadenopathy. Rebiopsy of the rectal wall had similarly distributed viral inclusions that positive for HSV IHC and negative for malignancy. The patient was prescribed valacyclovir, however was noncompliant with the medication. Two months later, he presented again with continuing severe rectal pain and unchanged findings on imaging. Following treatment with valacyclovir for two weeks, the pain resolved.

## 4. Discussion

Herpes simplex virus infection is commonly seen in HIV-positive and MSM patients [1]. We revisit HSV infection of the gastrointestinal tract and report two cases of male patients with HSV proctitis mimicking rectal malignancy clinically. One patient was MSM, and the other was HIV-positive. In these cases, there was a high clinical suspicion for rectal adenocarcinoma, given the mass-like rectal wall thickening several centimeters proximal to the anus, and the prominent associated lymphadenopathy seen on CT. Other STI were ruled out with laboratory testing in both patients. Both patients underwent rectal biopsies. One patient had a concomitant positive HSV culture. Both patients had resolution of symptoms following antiviral treatment for HSV infection.

Histologically, both the rectal biopsies in our cases were composed predominantly of ulcer and granulation tissue with mixed inflammatory infiltrate composed of prominent lymphocytes, plasma cells, and eosinophils, along with necrosis and fragments of rectal mucosa with mucosal prolapse and reactive changes. Careful examination of the granulation tissue in both cases showed viral cytopathic inclusions suggestive of HSV infection including subtle multinucleation, nuclear molding, and margination of chromatin within endothelial cells of only few vessels. HSV IHC confirmed the diagnosis of HSV by highlighting viral inclusions infecting endothelial cell nuclei of rare vessels within the granulation tissue, and in one case, HSV IHC also highlighted rare clusters of granulation tissue fibroblast nuclei. CMV IHC was negative in both cases. No other infectious organisms were identified in either biopsy. No perianal squamous epithelium was present in the original biopsies.

To our knowledge, there are four additional reported cases of HSV proctitis presenting as rectal pseudotumors that were biopsied, reported in the medical literature [9–12]. Similar to our patients, three of the previously reported cases were male patients and one female, the majority of whom were HIV positive. All had a mass-like lesion present proximal to the dentate line, and most responded to antiviral treatment. Chen et al. described the presence of a pseudolymphomatous infiltrate in the background tissue of their case, which was not present in our cases, and Ayoade et al. reported positive HSV-1 IHC in rectal epithelial cells, which was not seen in our cases. Charles et al. described inflammation with syncitia formation, with no viral inclusions on biopsy; however, HSV-2 PCR was positive on the tissue. Similar to our cases, HSV viral inclusions within endothelial cell nuclei were described in the case reported by Bai et al. While Chen et al. reported prominent fibroblast nuclei, none of the other reported cases described viral inclusions within scattered fibroblast nuclei, as we observed in one of our cases. Squamous epithelium was not present in our cases, nor was it described in any of the above-reported cases. In addition, Hughes and Lewis recently reported a clinical case of a 30-year-old MSM with nonulcerative proctitis and no perianal lesions who was empirically treated for chlamydial and gonorrhoeal infection with no improvement. Forty-eight hours later, a rectal swab from the patient was positive for HSV-2. The patient was recalled and treated with valacyclovir, and symptoms regressed. The authors highlight that rectal HSV can occur without the presence of perianal lesions [13].

In conclusion, we describe the histologic, immunohistochemical, and clinical features of HSV proctitis of the proximal rectum clinically mimicking rectal adenocarcinoma in two male patients presenting with rectal masses several centimeters above the anal verge. The presentation of anogenital HSV infection is more commonly seen in the squamous epithelium of the anorectal canal/perianal skin at the edge of an ulcer, rather than in the more proximal portion of the rectum as seen in our cases. HSV inclusions within endothelial cell and fibroblast nuclei in a rectal biopsy could be easily missed if the pathologist is not aware of this unusual distribution of HSV inclusions, and HSV is not considered in the differential diagnosis. Ancillary studies including HSV IHC and viral culture can be helpful to confirm the diagnosis and lead to appropriate and timely antiviral therapy in these patients.
